# Visualizing the Central Nervous System: Imaging Tools for Multiple Sclerosis and Neuromyelitis Optica Spectrum Disorders

**DOI:** 10.3389/fneur.2020.00450

**Published:** 2020-06-17

**Authors:** Joseph Kuchling, Friedemann Paul

**Affiliations:** ^1^Experimental and Clinical Research Center, Max Delbrück Center for Molecular Medicine, Charité-Universitätsmedizin Berlin, Corporate Member of Freie Universität Berlin, Humboldt–Universität zu Berlin, and Berlin Institute of Health, Berlin, Germany; ^2^NeuroCure Clinical Research Center, Charité–Universitätsmedizin Berlin, Corporate Member of Freie Universität Berlin, Humboldt–Universität zu Berlin, and Berlin Institute of Health, Berlin, Germany; ^3^Department of Neurology, Charité–Universitätsmedizin Berlin, Corporate Member of Freie Universität Berlin, Humboldt–Universität zu Berlin, and Berlin Institute of Health, Berlin, Germany; ^4^Berlin Institute of Health, Berlin, Germany

**Keywords:** multiple sclerosis, neuromyelitis optica spectrum disorders (NMOSD), magnetic resonance imaging, optical coherence tomography, neuroimaging

## Abstract

Multiple sclerosis (MS) and neuromyelitis optica spectrum disorders (NMOSD) are autoimmune central nervous system conditions with increasing incidence and prevalence. While MS is the most frequent inflammatory CNS disorder in young adults, NMOSD is a rare disease, that is pathogenetically distinct from MS, and accounts for approximately 1% of demyelinating disorders, with the relative proportion within the demyelinating CNS diseases varying widely among different races and regions. Most immunomodulatory drugs used in MS are inefficacious or even harmful in NMOSD, emphasizing the need for a timely and accurate diagnosis and distinction from MS. Despite distinct immunopathology and differences in disease course and severity there might be considerable overlap in clinical and imaging findings, posing a diagnostic challenge for managing neurologists. Differential diagnosis is facilitated by positive serology for AQP4-antibodies (AQP4-ab) in NMOSD, but might be difficult in seronegative cases. Imaging of the brain, optic nerve, retina and spinal cord is of paramount importance when managing patients with autoimmune CNS conditions. Once a diagnosis has been established, imaging techniques are often deployed at regular intervals over the disease course as surrogate measures for disease activity and progression and to surveil treatment effects. While the application of some imaging modalities for monitoring of disease course was established decades ago in MS, the situation is unclear in NMOSD where work on longitudinal imaging findings and their association with clinical disability is scant. Moreover, as long-term disability is mostly attack-related in NMOSD and does not stem from insidious progression as in MS, regular follow-up imaging might not be useful in the absence of clinical events. However, with accumulating evidence for covert tissue alteration in NMOSD and with the advent of approved immunotherapies the role of imaging in the management of NMOSD may be reconsidered. By contrast, MS management still faces the challenge of implementing imaging techniques that are capable of monitoring progressive tissue loss in clinical trials and cohort studies into treatment algorithms for individual patients. This article reviews the current status of imaging research in MS and NMOSD with an emphasis on emerging modalities that have the potential to be implemented in clinical practice.

## Introduction

Multiple sclerosis (MS) and neuromyelitis optica spectrum disorders (NMOSD) are inflammatory, autoimmune central nervous system conditions that have shown increasing incidence and prevalence over the past decades (1–5). While MS is the most frequent inflammatory CNS disorder in young adults, NMOSD is a rare disease. Relative frequency within the demyelinating CNS diseases varies widely among different ethnicities and regions, accounting for ~1% of demyelinating disorders (6, 7). Based on results from population-based studies, NMOSD prevalence broadly ranges from 0.52 to 7.7 per 100,000 (7). Although NMOSD frequency in Asian and White/Caucasian ethnicities seems to be comparably similar (4, 8), Blacks seem to have highest NMOSD prevalence of up to 13/100,000 as inferred from mixed Northern American populations (9, 10).

For a long time, NMOSD had been seen as a rare variant of MS; however, the seminal discovery of a highly specific serum IgG autoantibody to the astrocyte water channel aquaporin-4 (AQP4) in up to 80% of NMOSD patients and subsequent research into the role of these antibodies in disease pathogenesis and lesion formation has made clear that this is a condition distinct from MS (11–17). Clinical experience has then shown that most immunomodulatory drugs used in MS are inefficacious or even harmful in NMOSD, emphasizing the need for a timely and accurate diagnosis and distinction from MS (18–21). Despite distinct immunopathology and differences in disease course and severity, there might be considerable overlap in clinical and imaging findings, posing a diagnostic challenge for managing neurologists. Differential diagnosis is facilitated in case of a positive serology for AQP4-abs obtained with a highly specific cell-based assay but might be difficult in seronegative cases or when less specific assays for AQP4-abs are used (22, 23).

Imaging of the brain, optic nerve, retina, and spinal cord is a procedure of paramount importance when managing patients with inflammatory CNS conditions at first presentation to enable diagnosis and differential diagnosis (24–28). Once a diagnosis has been established, imaging techniques are often deployed at regular intervals over the disease course as surrogate measures for disease activity and progression and to surveil treatment effects (29, 30). Although the application of some imaging modalities for monitoring of disease course was established decades ago in MS, the situation is less clear in NMOSD in which work on longitudinal imaging findings and their association with clinical disability is scant (26). Moreover, as long-term disability is mostly attack-related in NMOSD and does not stem from insidious progression as in MS, regular follow-up imaging might not be useful in the absence of clinical events. However, with accumulating evidence for covert tissue alteration in NMOSD and with the advent of approved immunotherapies, the role of imaging in the management of NMOSD might have to be reconsidered in the near future (31–37). In addition, imaging markers indicating impending relapses are an unmet need in NMOSD. On the contrary, MS management still faces the challenge of implementing imaging techniques that are capable of monitoring progressive tissue loss (for example brain or spinal cord atrophy) in clinical trials and cohort studies into treatment algorithms for individual patients (38–40).

This article reviews the current status of imaging research in MS and NMOSD with an emphasis on emerging modalities that have the potential to be implemented in clinical practice for diagnosis, differential diagnosis, and monitoring of disease course and immunotherapies.

## Multiple Sclerosis

As in previous versions of the MS diagnostic criteria, conventional MRI of the brain and spinal cord (T2/Flair/T1 post gadolinium sequences) is a cornerstone for an MS diagnosis within the 2017 revision of the McDonald criteria (41, 42), taking potential “red flags” and “MS mimics” into consideration that may point to an alternative diagnosis (24, 25). However, sensitivity of the 2017 criteria might have improved, and time to diagnosis appears to be shorter at the expense of specificity (43–45). Thus, frequent misdiagnosis of MS based upon misinterpretation of imaging findings on conventional MRI in conjunction with atypical clinical presentations even by MS experts has remained an alarming issue (46–49).

Recently the so-called “central vein sign” (CVS) was proposed as a potential new biomarker for a more specific MS diagnosis, emerging from observations, mostly at ultra-high field MRI studies, that MS lesions are frequently characterized by a small intralesional vein in contrast to relevant imaging differential diagnoses, such as NMOSD, small vessel disease, inflammatory CNS vasculopathies, Susac syndrome, and others (50–56).

**Figure 1:**
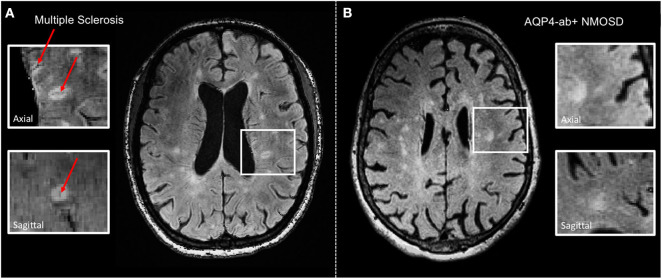
Representative axial 3 T FLAIR-SWI images from individuals with **(A)** relapsing–remitting multiple sclerosis (RRMS; 28-year-old woman) and **(B)** AQP4-antibody-positive neuromyelitis optica spectrum disorder (AQP4+-NMOSD; 76-year-old woman). The central vein sign *(red arrows)* is present in the majority of MS lesions but not in white matter lesions in NMOSD. White boxes show magnified views of lesions in axial and sagittal plane. T, Tesla; FLAIR, fluid-attenuated inversion recovery; SWI, susceptibility-weighted imaging; RRMS, relapsing-remitting multiple sclerosis; AQP4-ab+, AQP4-antibody positive; NMOSD, neuromyelitis optica spectrum disorder.

CVS is now reliably assessable at 3T, for example, using T2^*^/FLAIR and co-registered SWI images, and might, therefore, become a clinically applicable imaging feature to discriminate MS from classical mimics at a high specificity (56–58) (Figure 1). In one study, a threshold of 50% perivenular lesions discriminated MS from inflammatory vasculopathies, such as Behcet disease, primary angiitis of the CNS, antiphospholipid syndrome, Sjögren syndrome, and systemic lupus erythematosus (SLE), with 100% accuracy (56), and another multicenter study conducted by the MAGNIMS consortium reported a specificity of 83% for a 35% CVS proportion threshold for discriminating MS from mimics such as NMOSD, SLE, migraine, cluster headache, diabetes, and other types of small vessel disease (58). Perhaps less onerous in the clinical situation is the three-lesion CVS criterion, which yielded a specificity of 89% for discriminating MS from other conditions. In this study, sensitivity was better with an optimized T2^*^-weighted sequence. These findings require replication in prospective studies enrolling patients with various ethnic backgrounds and from different regions of the world and will hopefully lead to a novel imaging biomarker with high specificity for MS that might find its way into a future revision of the McDonald criteria.

**Figure 2:**
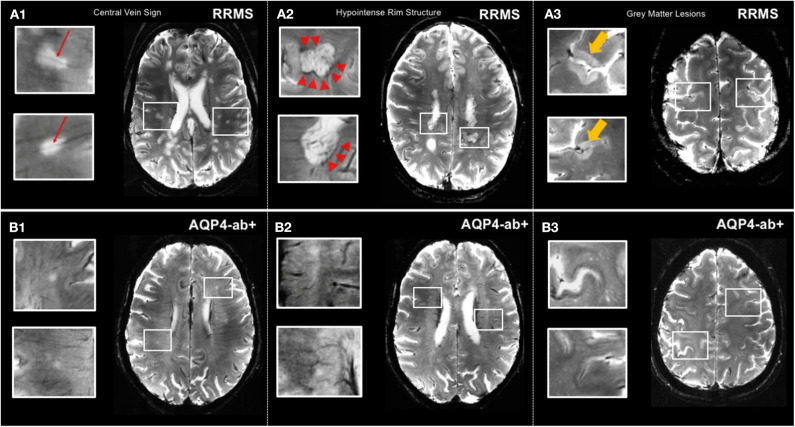
MS-specific 7 T MR imaging markers displayed by T2*-weighted sequence. **(A1)** Lesions in relapsing-remitting MS commonly exhibit a central vein *(red arrows)*. **(A2)** Hypointense rim structures *(red arrow-heads)* are prevalent in a subset of MS lesions. **(A3)** 7 T MRI allows for the delineation of gray matter lesions in great detail. **(B1,B2)** Central vein sign and hypointense rim structures are absent in lesions of AQP4+-NMOSD patients. **(B3)** Gray matter lesions are commonly absent in AQP4+-NMOSD. MS, multiple sclerosis; T, Tesla; FLAIR, fluid-attenuated inversion recovery; SWI, susceptibility-weighted imaging; RRMS, relapsing-remitting multiple sclerosis; AQP4-ab+, AQP4-antibody positive; NMOSD, neuromyelitis optica spectrum disorder. LGN, lateral geniculate nucleus; V1, primary visual cortex.

Although MRI T2 hyperintense lesions represent one of the major diagnostic hallmarks of MS, macroscopic MRI-visible lesions are commonly termed as “tip of the iceberg” because many more lesions are detected by histopathology at a microscopic level (59). Particularly, cortical lesions are widely elusive to conventional MRI at 3 Tesla although introduction of ultra-high field 7 T MRI more than doubles detection of cortical MS lesions (60) (Figure 2). Of note, post mortem studies showed that sensitivity to detect cortical lesions at 7 T is strongly influenced by their histopathological subtype, ranging from 11 to 100% (61). Hence, cortical pathology still remains more extensive than even 7 T MRI can reveal.

Cortical lesions are considered a distinctive feature of MS and are rarely present or even totally absent in other conditions mimicking multiple sclerosis, such as migraine or NMOSD (60). Intriguingly, presence and number of cortical pathology appears to correlate with clinical outcomes, most notably cognitive impairment in MS (62). However, clinical significance of cortical lesions is controversially discussed throughout the literature, and further 7 T MR studies, including investigations with improved visualization at magnetization-prepared 2 rapid acquisition gradient echoes (MP2RAGE), are highly warranted to clarify potential diagnostic and prognostic value of MS cortical pathology (63).

Brain and spinal cord volumetric imaging is another MR-based measure that might have the potential to be used in clinical practice to monitor disease progression and treatment response. Both neuropathology and imaging studies have shown that atrophy of the entire brain, including cortical and deep gray matter (DGM) as well as the spinal cord, are typical hallmarks of MS from earliest disease stages (64–68) and that, particularly, cerebral gray matter volume loss (above all, the deep gray matter) and spinal cord atrophy correlate with clinical disability and cognitive impairment and are predictive of further disease progression in longitudinal studies (69–78). In clinically stable and untreated MS patients, annual brain volume loss ranges from ~0.5 to 1.0% in comparison to 0.1–0.3% for healthy subjects (73, 79). In a recent large European multicenter study comprising more than 1,200 patients with MS and more than 200 healthy subjects, volumes of deep and cortical gray and white matter were obtained, and participants followed over an average of 2.41 years (69). Deep gray matter showed the fastest annual atrophy rates, which ranged from −1.34 to −1.66% in various MS forms and was −0.88% in CIS and −0.94% in HC. Of all regional volumes quantified at baseline, only deep gray matter volume predicted time to EDSS progression, which underscores the relevance of DGM loss for disability accumulation. A 7.5-year longitudinal study (range 1–12 years), 206 MS patients and 35 healthy controls reported a cutoff of −0.4% annualized brain volume change to have a sensitivity of 65% and a specificity of 80% for discriminating physiological from pathological brain volume loss (80). The clinical relevance of this cutoff remains to be demonstrated. Various immunotherapies have been shown to decelerate brain volume loss; however, it is currently unclear how this observation would inform treatment decisions in individual patients (81–85). Brain volumetric measurements for use in individual patients are still hampered by numerous technical challenges, such as inter-session variability, influence of physiological factors (for example, hydration status), normal aging and comorbidities on brain volumes, time of day of MR scan, effect of lesion filling on post-acquisition quantitation procedures, and systematic differences pertaining to scanners and sequences parameters (38, 86). Therefore, despite sufficient accuracy of brain volume measurements in observational and interventional cohort studies, the technology is not yet apt to reliably investigate changes in individual patients within periods of less than a few years and therefore—also in light of the various physiological sources of error—atrophy measurements are currently not usable to monitor therapy in MS (30, 73, 87). Besides technical advances to reduce measurement variability, a better understanding into the neuropathological correlates and drivers of deep and cortical gray matter atrophy and whole brain volume loss is urgently required (38). The same applies to spinal cord atrophy, which is relatively easy to measure at the cervical level (mean upper cervical cord area or MUCCA) even on brain scans that cover the superior part of the spinal cord down to the C2/C3 level (88). However, physiological fluctuations and change over time of this measure in healthy subjects are unknown, and although some studies have reported spinal cord atrophy rates of between <0.5% and more than 2% per year, with progressive and clinically deteriorating patients exhibiting faster atrophy rates, it is not established how MUCCA could be used to monitor individual patients (88–93). However, a recent study suggests that conventional measures of spinal cord involvement, such as focal lesions and emergence of new lesions, can be used to estimate risk of secondary progressive MS and EDSS at 15 years in patients with clinically isolated syndrome (94).

**Figure 3:**
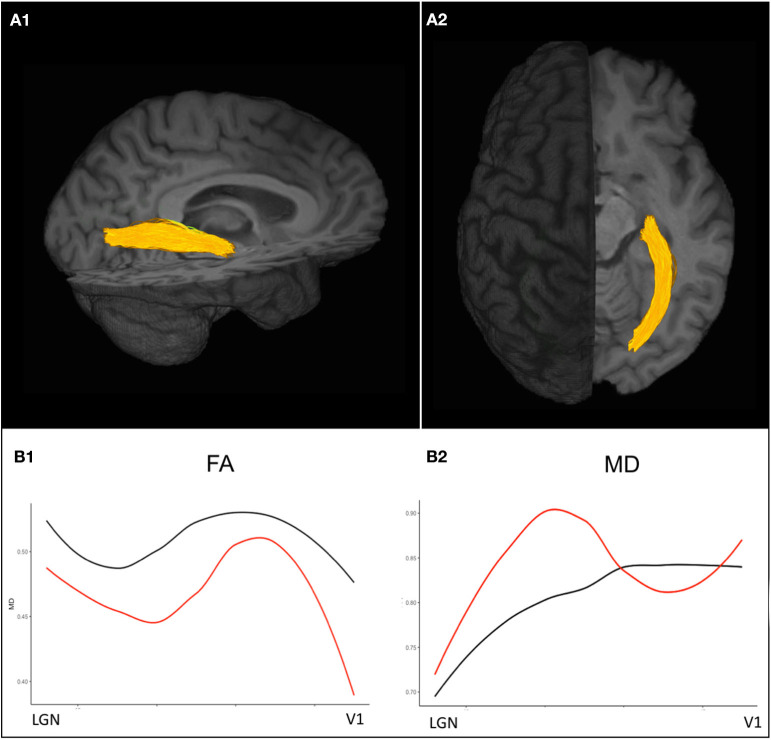
Diffusion-weighted imaging based probabilistic tractography allows for the delineation of the optic radiations displayed in **(A1)** sagittal and **(A2)** axial view. **(B1)** Diffusion tensor imaging (DTI) values along the optic radiation of an exemplary ON patient 3 years after attack *(red)* show decreased FA values compared to a healthy control *(black)* indicating trans-synaptic neurodegeneration after ON. **(B2)** MD values are pathologically increased in an exemplary ON patient almost throughout the entire course of the optic radiations compared to the exemplary healthy control. ON, optic neuritis; FA, fractional anisotropy; MD, mean diffusivity.

Other advanced MRI techniques have been recently applied to investigate pathogenetic processes associated with neurodegeneration and disease progression. Amid other emerging quantitative MRI approaches, diffusion tensor imaging (DTI), which relies on the detection of changes in the random translational motion of water molecules and thereby estimates the level of tissue degradation in the normal-appearing white matter, provides promising imaging markers to detect neuronal damage (83). Post mortem investigations showed fractional anisotropy (FA) decrease to be to associated with axonal loss and myelin density, thereby suggesting DTI FA to be a useful indicator of both neurodegeneration and demyelination in MS (95) (Figure 3). However, future histopathological and clinical studies on quantitative MR markers are highly warranted to validate the capacity of modern MRI in detecting and monitoring neurodegenerative MS pathology that remains elusive to conventional structural MRI.

In clinical management, the use of MRI to monitor treatment response still relies on conventional parameters, such as new or enlarging T2 lesions and gadolinium-enhancing lesions in conjunction with clinical measures (relapses, disability progression), measures summarized under the term “NEDA” (no evidence of disease activity) (30). However, NEDA seems to be a questionable treatment goal given that, in real-world observational studies, <10% of patients retain a NEDA status after more than 5 years (96), and even with highly effective immunotherapies, NEDA rates hardly exceed 50% (97). Moreover, the clinical relevance of this composite score has been called into question, for example, by data from a large prospective observational study with more than 500 MS patients from California showing that meeting the NEDA status at 2 years was not predictive of long-term stability (98). In addition, the NEDA concept has been heavily criticized because of ignoring other relevant and disabling symptoms of the disease, such as fatigue, cognitive problems, sleep disorders, depression, etc. (71, 99–109). Moreover, recent safety concerns as to the deposition of gadolinium-based contrast agents (predominantly linear compounds) in the dentate nucleus and other brain regions provide arguments against their frequent use in monitoring radiographic disease activity in otherwise stable patients (110–113). For detection of new brain lesions, a T2/FLAIR sequence is sufficient as long as rigorous standardization of image acquisition to ensure maximum comparability is guaranteed (29). To overcome the shortcomings and downsides of the current NEDA concept, a new term (“minimal evidence of disease activity” or MEDA) has been proposed as well as a more sophisticated approach to monitor MS therapy taking also patient-reported outcomes into consideration (“multiple sclerosis decision model” or MDSM) (114, 115). However, both concepts lack prospective validation, so their use in clinical management cannot be unambiguously recommended. The same applies to the upgraded NEDA concept that includes brain atrophy into the composite measure (NEDA-4) (116, 117).

Numerous non-conventional and advanced imaging modalities are currently under investigation that may help improve visualization and quantification of (covert) tissue damage in the gray and white matter of the brain and the spinal cord and could be used as an imaging surrogate of remyelination and repair; among them are magnetization transfer imaging, diffusion tensor imaging, myelin water imaging, susceptibility weighted imaging, magnetic resonance spectroscopy, sodium imaging, PET imaging, ultra-high field imaging at 7 Tesla, functional imaging with resting state fMRI, T1/T2-weighted ratio calculable from conventional T1- and T2-weighted images, machine-learning based imaging, magnetic resonance elastography, and several others, none of which will probably be used in clinical practice in the near future (27, 52, 83, 88, 118–135). Nonetheless, these endeavors are important to deepen our understanding of mechanisms of tissue damage in MS and to devise better imaging endpoints for clinical trials and routine care than those currently in use.

A recent emerging imaging tool in neuroinflammation is retinal optical coherence tomography (OCT), a technique that takes advantage of the retinal backscatter and reflection of low coherent light and enables the reconstruction of structural images of the various retinal layers with a resolution of a few microns and a very time-efficient image acquisition of only a few minutes in a cooperative patient (136).

OCT has been used for more than a decade in clinical neuroimmunology, mostly in cohort studies and occasionally as an endpoint in acute optic neuritis trials (137–139), and it is at the verge of entering clinical management of patients with MS and related disorders. Most widely used retinal measures of neuro-axonal damage are the (peripapillary) retinal nerve fiber layer (pRNFL) and the ganglion cell layer (GCL) that is often reported together with the inner plexiform layer (IPL) due to inaccuracy of segmentation and then displayed as ganglion cell/inner plexiform layer (GCIPL) (39, 136). In MS, OCT has been shown to be reliably applicable in a multicenter setting (140). Certain standards for quality control of OCT scans and reporting of data have been proposed, and confounders, for example, the influence of retinal vessels on neuroaxonal measures, have to be taken into consideration (141–143). Thinning of the RNFL or the GCL/GCIPL are consistently reduced according to a high number of studies both in MS eyes with a history of prior optic neuritis as well as to a lesser extent in MS eyes without prior ON (144–146). Retinal thinning in MS is detectable from the earliest disease stages (147, 148) and is associated with altered visual function, visual quality of life, VEP latencies, overall disability, cognitive performance, inflammatory brain lesions, and both spinal cord and brain atrophy, and has been shown to reflect clinical and radiographic disease activity in longitudinal studies (149–162). A recent meta-analysis comprising more than 1,000 eyes calculated an average pRNFL loss of 20 μm in eyes with prior ON and of 7 μm in eyes without history of ON (NON), and average GCIPL thinning was 16 μm in ON eyes and 6 μm in NON eyes (144). Annual rates of RNFL thinning in longitudinal studies range from ~0.2 to 2.0 μm per year and depend on disease stage and treatment status. In general, patients with progressive MS tend to show more severe retinal thinning than RRMS patients (145). A retrospective, non-randomized “real-world” study suggested that MS immunotherapies may differentially affect the rate of annual ganglion cell loss with faster thinning in patients treated with interferon beta or glatiramer acetate and slower thinning in patients on natalizumab (163). In a longitudinal monocenter study in 72 patients with MS from Italy, NEDA status was associated with relatively preserved RNFL over 2 years; patients with NEDA (32% of the cohort) had an average RNFL loss of −0.93 μm as compared to −2.83 μm in the evidence of disease activity (EDA) group (164). Patients with stable EDSS over the course of the study had on average a RNFL loss of −1.33 μm as in contrast to −3.23 μm in patients with an EDSS worsening of ≥0.5 points. A cutoff of −1.25 μm RNFL loss was able to classify the NEDA status with a sensitivity of 80% and a specificity of 81.4%. A large retrospective multicenter study conducted by the International Multiple Sclerosis Visual System Consortium (www.imsvisual.org) in 879 patients with various stages of MS suggests that pRNFL may be used to predict disability worsening (165). Patients with a pRNFL below 92/93 μm (different OCT machines used) had a 60% increased risk of disability progression after 1 year, and those with a pRNFL <87/88 μm had a 4-fold increased risk of progression on the EDSS after 4–5 years.

Another retrospective study in 305 MS patients in different stages of the disease and with a median interval of 7.9 years from the acquisition of an OCT scan (using the older time domain technology to measure the pRNFL) (166) to the last EDSS assessment evaluated the relationship between both parameters (167). Each 1 μm decrease in the baseline pRNFL was associated with an increase in EDSS of 0.024 points, suggesting that a pRNFL measurement may help to prognosticate disability within 6–9 years later. Similar results were obtained when adjusting for the presence of previous optic neuritis episodes.

Also in a clinically isolated syndrome (CIS) scenario OCT may be helpful to assess the risk of further disease activity. A bicenter study from Germany grouped 89 patients with a CIS as a qualifying event into three groups according to their baseline GCIPL values in NON eyes (168). Patients in the lowest tertile (ranging from 58.7 to 69.2 μm) had a hazard ratio of 3.43 for not meeting NEDA status within the follow-up period (max 2.5 years) as compared to patients in the highest GCIPL tertile (ranging from 74.2 to 84.8 μm). In contrast, other established predictors of further disease activity in CIS patients, such as MRI T2 lesion load, sex, or ON as a qualifying symptom, were not predictive of a subsequent NEDA status. For the most recent revision of the McDonald criteria it was controversially discussed whether affection of the visual system should be used to demonstrate dissemination in space or time. However, “the panel felt the data … were insufficient to support incorporation into the McDonald criteria” but “studies to validate MRI, visual evoked potentials, or optical coherence tomography in fulfilling DIS or DIT in support of a multiple sclerosis diagnosis were identified as a high priority.” (41). A first step toward this direction has been undertaken by the IMSVISUAL Consortium that recently pooled data from more than 1,500 patients with MS to determine the optimal intereye differences in RNFL and GCIPL thicknesses for identifying unilateral optic nerve lesions defined as history of acute unilateral optic neuritis (169). Using receiver-operating characteristic curve analysis, an intereye difference of 5 μm for RNFL and of 4 μm for GCIPL was demonstrated as an optimal threshold for identifying unilateral optic nerve lesions. Eighteen percent of patients in the entire cohort had intereye differences of >5 μm for RNFL and 12% of >4 μm for GCIPL without history of acute ON. In line with another recent study (170), these findings suggest that these measures may complement MRI to demonstrate dissemination in space and time.

## Neuromyelitis Optica Spectrum Disorders (NMOSD)

In 2015, new diagnostic criteria for NMOSD with and without (or with unknown) AQP4 antibodies have been proposed against the background of a broadening clinical spectrum that was recognized with the increasing number of patients tested for AQP4 antibodies (171). Imaging features regarded as characteristic yet not pathognomonic for NMOSD are a core element of the 2015 IPND criteria, in particular in seronegative patients or in subjects with unknown AQP4 ab status. The main goal of listing these imaging findings is to help clinicians discriminate NMOSD from other conditions, namely MS, and thus reduce the chance of misdiagnosis. Imaging abnormalities in NMOSD are described according to the anatomical location in the brain, optic nerve, and spinal cord. The establishment of the 2015 IPND criteria have led to a rise in the number of diagnosed NMOSD cases by up to 76%, and fortunately, diagnostic delay was considerably decreased from 53 months by the 2006 criteria to 11 months by the 2015 criteria (172, 173).

Over the past 15 years, an impressive number of imaging studies have made clear that—in contrast to earlier views—most NMOSD patients exhibit some kind of brain lesions. Lesions are not always located in areas of high AQP4 expression, and a considerable proportion (42%) may even meet Barkhof criteria for multiple sclerosis (24, 26, 174, 175). Most studies in NMOSD have used conventional MR sequences; non-conventional and advanced imaging studies are scant and have mostly yielded inconsistent results (176).

According to newer studies, the majority of NMOSD patients show some kind of brain lesions although findings considered highly suggestive and suspicious of an NMOSD diagnosis are less prevalent. Between 43 and 70% of NMOSD patients have brain lesions at onset, and up to 85% of patients meeting the 2006 Wingerchuk criteria for NMO and up to 89% of seropositive patients were reported to have brain abnormalities (12, 26, 177–180). Brain lesions considered highly suggestive of NMOSD are diencephalic lesions surrounding the third ventricle and cerebral aqueduct, which are often asymptomatic but may occasionally present with inappropriate antidiuretic hormone secretion, narcolepsy, hypothermia, hypotension, or hyperprolactinemia. Another very characteristic predilection site is the dorsal brainstem: Lesions adjacent to the fourth ventricle, including the area postrema and the nucleus tractus solitarii, are highly specific for NMOSD, reported in 7–46% of patients with NMO (26, 181). The typical clinical manifestation is with intractable hiccups, nausea, and vomiting (171). Lesions in the corpus callosum (CC) have been described in 12–40% of patients with NMOSD. Although the location in the CC is not a unique finding that differentiates NMOSD from MS, NMOSD callosal lesions are in contrast to MS located immediately next to the ventricles and follow the ependymal lining (26). CC lesions may extend into the cerebral hemisphere, forming an extensive and confluent white matter lesion. Acute CC lesions are often edematous and heterogeneous with a “marbled pattern” (182). Hemispheric white matter lesions may appear extensive and confluent, are often tumefactive (>3 cm in longest diameter), or have a long spindle-like or radial shape following white matter tracts; they usually have no mass effect. They may occasionally mimic posterior reversible encephalopathy syndrome (PRES) or Baló-like lesions or may resemble acute disseminated encephalomyelitis (ADEM) or CNS malignancies and were reported to be more frequent in AQP4 ab seropositive than seronegative patients (26). Hemispheric white matter lesions may disappear but may also remain as cyst-like or cavitary changes. Also corticospinal tracts may be involved in NMOSD with either unilateral or bilateral involvement and were reported in up to 44% of patients. These lesions may extend from the deep white matter in the cerebral hemisphere through the posterior limb of the internal capsule to the cerebral peduncles of the midbrain or pons. They are often contiguous, longitudinally extensive, and may follow pyramidal tracts. The reason for involvement in NMOSD is unclear as corticospinal tracts are not areas of high AQP4 expression. The probably most frequent type of brain lesions in NMOSD reported in up to 84% of patients are “non-specific” lesions: punctate or small (<3 mm) dots or patches of hyperintensities on T2-weighted or FLAIR sequences in the subcortical or deep white matter that are usually asymptomatic and tend to increase with age, presumably owing to age-related vascular comorbidities. These lesions may nonetheless pose diagnostic challenges vs. MS and other conditions. Few studies have looked into gadolinium-enhancing brain lesions in NMOSD; up to 36% of patients have shown enhancing lesions that are often poorly marginated, subtle, or show a patchy pattern. One study from Japan suggested “cloud-like enhancement” to be a characteristic enhancement pattern in NMOSD (178). Nodular enhancement or meningeal enhancement have also been described, and linear enhancement of the ependymal surface of the lateral ventricles (“pencil-thin lesion”) was proposed as another imaging feature characteristic of NMOSD (183, 184).

In contrast to MS, cortical lesions are usually absent in NMOSD, which is supported by 3 T double inversion recovery and ultra-high field MR studies that investigated the cortex in NMO as well as by several histopathologic studies (54, 185, 186) (Figure 2). Additionally, a lower proportion of NMOSD lesions show the CVS or display hypointense rims compared to lesions in MS (54, 57) (Figure 2). The challenging overlap of brain lesion occurrence and numbers between NMOSD and MS have prompted the use of algorithmic approaches to improve differential diagnosis. For example, one study from the UK in 26 AQP4 ab seropositive NMOSD (63% of whom had brain T2 lesions and 16% met Barkhof criteria) and 50 RRMS patients replicated a few key features in both conditions that appeared to be discriminative, among them a smaller lesion size and fewer numbers in NMOSD as compared to MS, and MS exhibited a greater coherence of lesion location (most likely to occur adjacent to the posterior of the body of the lateral ventricle in the parietal white matter) (187). In contrast, the lesional region with the greatest likelihood to be within the NMOSD group and not the MS group was adjacent to the fourth ventricle in the pons. Both groups had callosal lesions, but NMOSD patients showed no U fiber lesions and no Dawson's fingers. A combination of morphologic and locational criteria (at least one lesion adjacent to the body of the lateral ventricle and in the inferior temporal lobe or the presence of a subcortical U fiber lesion or a Dawson's finger–type lesion) could distinguish patients with MS from those with NMOSD with 92% sensitivity, 96% specificity, 98% positive predictive value, and 86% negative predictive value (187).

Of note, previous research shows that non-lesional tissue damage as measured by non-conventional imaging, such as DTI, may not occur in NMOSD except in the connecting tracts upstream and downstream of lesions (26). Although these findings lend support to the notion that NMOSD, in contrast to MS, may be a lesion-dependent disease that produces relapses without more generalized neurodegenerative pathology, the presence of potential subclinical tissue alterations in NMOSD affecting the afferent visual system has been controversially discussed. Recent DTI investigations in NMOSD patients without a clinical history of visual pathway affection showed structural retinal damage and pathological optic radiation DTI FA decrease outside attack-related lesions, suggesting a presumptive AQP4-ab–related astrocytopathy (188). These findings are in accordance with histopathological studies reporting on astrocytic end feet changes within LETM lesions and spinal cord atrophy in AQP4-ab–positive patients without previous myelitis attacks (189). Yet the question as to whether neurodegenerative non-lesion-related pathology exists in NMOSD is still under debate and needs to be further elucidated by future combined *in vivo* and *ex vivo* MRI investigations.

**Figure 4:**
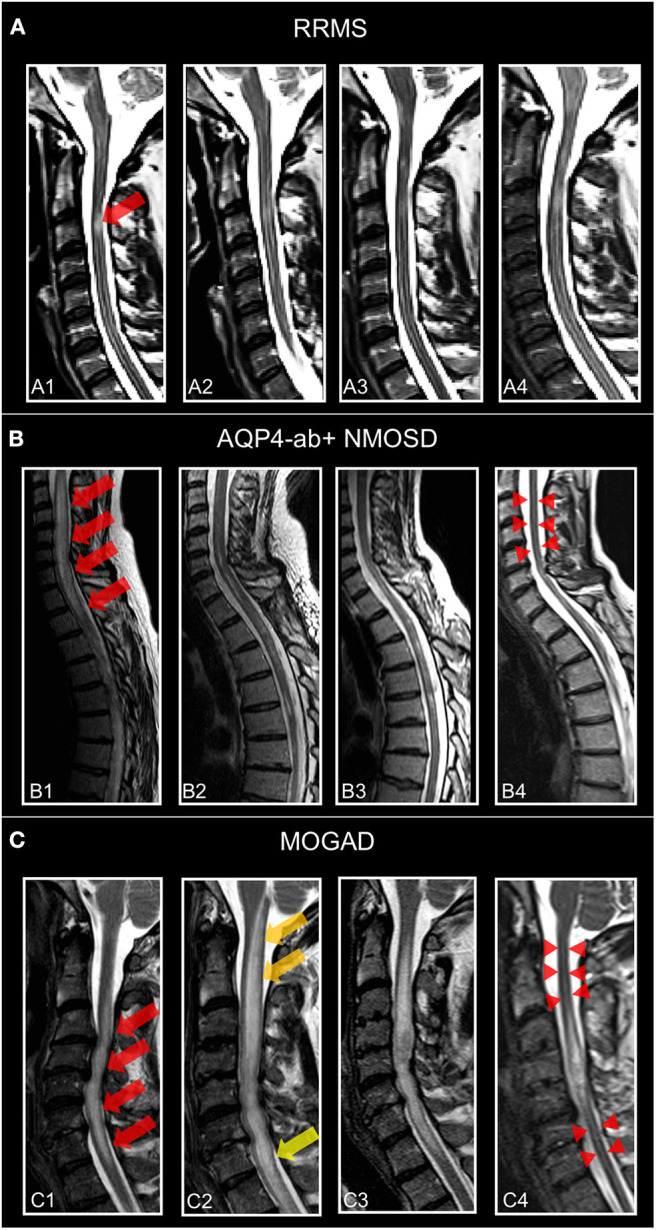
Representative T2-weighted spinal cord images from individuals. **(A)** Patient with relapsing–remitting multiple sclerosis (30-year-old woman) and MS-related myelitis and spinal cord imaging at **(A1)** 1 months, **(A2)** 2 months, **(A3)** 24 months, and **(A4)** 72 months after attack. Short extent (<3 segments) spinal cord lesion *(red arrow)* at C3 with typical morphology of MS-related myelitis. **(B)** Patient with AQP4-antibody-positive neuromyelitis optica spectrum disorder (36-year-old woman) and NMOSD-related LETM and spinal cord imaging at **(B1)** 2 months, **(B2)** 5 months, **(B3)** 12 months, and **(B4)** 60 months after attack. Spinal cord lesion *(red arrows)* with longitudinal morphology (C2-Th1; >3 segments) and subsequent atrophy *(red arrow-heads)* typical of NMOSD-related LETM. **(C)** Patient with MOG antibody associated disease (41-year-old woman) and MOGAD-related LETM and spinal cord imaging at **(C1)** 7 months, **(C2)** 8 months, **(C3)** 24 months, and **(C4)** 48 months after attack. Initial LETM (C3-C7; *red arrows)* with remarkable increase in length after relapse at month 8 **(C2)** *(yellow arrows)* and subsequent atrophy *(red arrow-heads)*. RRMS, relapsing-remitting multiple sclerosis; AQP4-ab+, AQP4-antibody positive; NMOSD, neuromyelitis optica spectrum disorder; LETM, longitudinally-extensive transverse myelitis; MOGAD, myelin-oligodendrocyte-glycoprotein associated disease.

Longitudinally extensive myelitis lesions (LETM) spanning three or more contiguous vertebral segments have long been regarded as an imaging feature highly suggestive of NMOSD (Figure 4). Sensitivity and specificity for this criterion were 98 and 83%, respectively, in the patient cohort underlying the 2006 Wingerchuk criteria (177). Long cord lesions occur more frequently in the cervical cord from which they may extend into the brainstem and the upper thoracic spinal cord than in lower cord regions. Moreover, NMOSD spinal cord lesions occupy more than half of the cord area and show preferential involvement in the spinal central gray matter during the acute and chronic stages of spinal cord inflammation. By contrast, the majority of MS spinal cord lesions are localized in the lateral and posterior white matter regions of the cord (190, 191). In the acute stage, spinal cord lesions often appear hypointense on T1 weighted scans (in contrast to MS); the inflamed cord is often swollen and may show patchy contrast enhancement. In the chronic stage, extensive cord atrophy with or without T2 signal changes may develop in NMOSD (Figure 4). It is important to bear in mind that the timing of the spinal MRI in relation to the onset of clinical symptoms may be crucial for the detection of longitudinally extensive cord lesions (192) and that ~15–20% of myelitis attacks in NMOSD may show short transverse myelitis lesions, spanning 2.5 vertebral segments or less (193, 194). This means that a spinal cord lesion shorter than three vertebral segments does not rule out an NMOSD diagnosis. Interestingly, a recent study on 91 Chinese NMOSD patients compared patients with LETM and patients with short transverse myelitis and showed that the latter suffered less motor and bowel or bladder disability and had minor EDSS at clinical onset but exhibited shorter time to relapse (195). Moreover, although extensive spinal cord lesions are highly suggestive of NMOSD, numerous other conditions have to be taken into consideration, such as sarcoidosis, spondylotic myelopathy, autoimmune GFAP astrocytopathy, neoplasms, lymphoma, spinal cord infarction, and many others (196–201). Recently, “bright spotty lesions” on T2 weighted sequences were reported to be a discriminative feature of NMOSD myelitis with specificity values up to 100% (202, 203).

Recently, a study in 48 NMOSD (all AQP4 ab positive), 22 MS patients, and 24 patients with other causes of LETM from the United States assessed spinal cord imaging features that may help discriminate NMOSD from MS. Four findings were found to be most distinctive of NMOSD vs. other etiologies: bright spotty lesions, T1 dark lesions, centrally located lesions, and lesions involving more than 50% of the cord area on axial sequences (190).

Another study in 116 NMOSD patients (98 AQP4 ab positive) found a high proportion of patients (49%) without typical NMOSD brain and spinal cord lesions and 37% meeting the 2010 McDonald criteria. Nonetheless, a combination of easily applicable criteria for brain and spinal cord images enabled distinction from matched MS patients with good sensitivity and specificity regardless of serostatus (204).

Although the optic nerve is frequently involved in NMOSD, few studies with orbital MRI have been conducted. AQP4 ab–positive NMOSD tends to show more often posterior involvement of the optic nerve(s) including the chiasm and a more frequent intracranial and bilateral affection of the optic nerve as compared to MS (205). In AQP4 ab–positive patients with ON, lesion length on orbital MRI in the acute phase was a strong predictor of visual outcome (206). Another study reported a longitudinally extensive optic nerve lesion exceeding 17.6 mm to have a sensitivity of 81% and a specificity of 77% for NMOSD vs. RRMS (207).

Advanced imaging with volumetric analyses, DTI, spectroscopy, and others have increasingly been performed over the past 10 years (176), albeit with many inconsistent results, presumably owing to small sample sizes, ethnic differences of the cohorts investigated, heterogeneity of the samples with regard to AQP4 ab serostatus, and others. It is, for example, still a matter of debate as to whether progressive brain volume loss occurs in NMOSD over time as is the case in MS and how different compartments, such as white matter, cortex, or deep gray matter, are differentially affected (208–218) and how this might be associated with often-overlooked and insufficiently treated symptoms, such as cognitive impairment and pain (219–222). Presence of occult white matter damage as measured, for example, by DTI, MTR, or T1 relaxation time, has also remained contentious, presumably again owing to differences in inclusion criteria and different approaches to the correction for multiple comparisons problem (223–228). Few functional imaging studies with resting state fMRI (rs-fMRI) in NMOSD suggest that visual impairment due to severe optic neuritis causes brain network connectivity changes, in particular in visual networks (229–232). The vast majority of MR spectroscopy studies of the brain has found no clear indication for covert white matter damage (233–237), and low myoinositol/creatine values in the lesional cervical cord of NMOSD patients suggest astrocytic damage (238).

Spinal cord atrophy and reductions of MUCCA are a consistent feature of AQP4 ab–positive NMOSD even in the absence of myelitis attacks/spinal cord lesions (209, 239, 240) (Figure 4). In one study in 27 NMOSD patients with a history of myelitis and six NMOSD without history of myelitis and without spinal cord lesions (all participants AQP4 ab positive), MUCCA was reduced in both groups vs. healthy controls and correlated with clinical disability (241). The clinical relevance of MUCCA to monitor disease activity and covert progression requires further studies.

**Figure 5:**
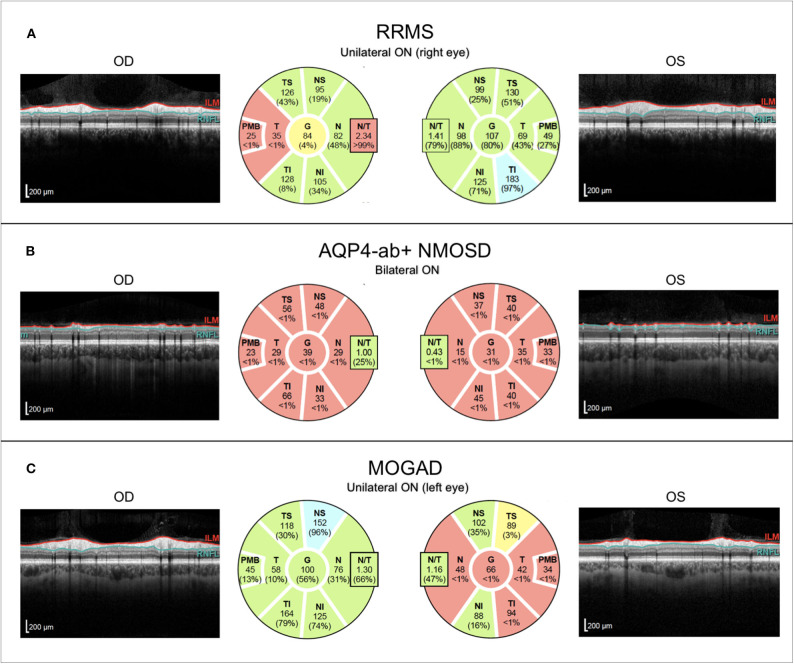
Representative OCT images from individuals with **(A)** relapsing–remitting multiple sclerosis with unilateral right-sided ON (RRMS; 41-year-old woman), **(B)** AQP4-antibody-positive neuromyelitis optica spectrum disorder with recurrent bilateral ON episodes (AQP4+-NMOSD; 25-year-old woman), and **(C)** MOG antibody associated disease with left-sided unilateral ON (MOGAD; 46-year-old man). OCT, optical coherence tomography; RRMS, relapsing-remitting multiple sclerosis; ON, optic neuritis; OD, right eye; OS, left eye; ILM, inner limiting membrane; RNFL, retinal nerve fiber layer; AQP4-ab+, AQP4-antibody positive; NMOSD, neuromyelitis optica spectrum disorder; MOGAD, myelin-oligodendrocyte-glycoprotein associated disease.

With the introduction of retinal OCT into clinical neuroimmunology, an increasing number of studies measuring retinal damage in NMOSD have been conducted over the past 10 years (242). Most studies have consistently shown that thinning of the RNFL and the GCIPL after an ON attack is on average more severe in AQP4 ab–positive NMOSD as compared to MS, a finding that aligns with the clinical experience of more severe vision loss in NMOSD (243–246). Impairment of visual quality of life caused by ON in NMOSD correlates with the extent of retinal damage measured by OCT, which underscores the potential clinical relevance of this technique (247) (Figure 5). Furthermore, this finding supports the strong recommendation for clinicians to treat ON as a neuroimmunological emergency as quickly and consequentially as possible because retinal ganglion cell loss starts early after clinical onset of symptoms, so timely administration of steroids or plasma exchange might help preserve retinal tissue and improve visual outcome (248–252). In NMOSD, ~25% of patients show so-called microcystic macular edema (MME) in the inner nuclear layer (INL) following ON, a frequency that is higher than in MS (5–10%) (242, 253–255). MME is not specific to NMOSD as it was described in a wide range of optic neuropathies. MME may be dynamic over time and seems to be associated with a less favorable visual outcome although neither its clinical relevance nor its pathophysiological underpinnings are entirely clear. Presumed mechanisms causing MME are vascular damage with extracellular fluid accumulation, Mueller cell pathology, and vitreous traction (242, 256, 257). A contentious issue in vision research in NMOSD is the occurrence of subclinical and progressive retinal thinning in NMOSD. In line with the clinical experience that disability is almost exclusively attack-related in NMOSD, some studies did not find progressive retinal thinning independent of ON (258). However, recent work has suggested that there is attack-independent ganglion cell loss in NMOSD—a finding whose clinical relevance needs to be further investigated (259). In addition, foveal changes have been detected in NMOSD patients without clinical evidence of optic neuritis of affection of the visual system, which suggests that AQP4 ab may directly target astrocytic Mueller cells in the retina, thus causing a primary retinal astrocytopathy (188, 260, 261). This finding is backed by animal work and human neuropathology data, both providing evidence for complement-independent AQP4 loss in Mueller cells and a retinal astrocytopathy (262, 263). Mathematical models to investigate the foveal shape will help investigate whether fovea changes may be used as a differential diagnostic feature for NMOSD and how these change over time in conjunction with functional visual outcomes (264).

In the past few years, a plethora of publications has reported on serum antibodies to myelin oligodendrocyte glycoprotein (MOG) in a subset of adult patients with an NMOSD phenotype (with optic neuritis being the most frequent clinical manifestation) and beyond (involvement of cranial and peripheral nerves and encephalopathy with seizures have been reported) using highly specific immunoassays (265–275). The current discussion evolves toward recognizing this condition as a disease entity distinct from AQP4 ab positive NMOSD and MS for which the acronyms “MOGAD” (MOG antibody associated disease) or “MOG-EM” (MOG antibody associated encephalomyelitis) were proposed (15, 276–279).

Neuroimaging studies with MRI in MOGAD are scant, and the few reports suggest that there is a broad overlap with AQP4 ab–positive NMOSD as to the presentation on conventional brain and spinal cord MRIs (279–284) although MOG patients were reported to show a more frequent involvement of the conus/lumbar spinal cord (285). From a clinical standpoint, it is important to bear in mind that up to 27% of patients with MOGAD may meet Barkhof criteria for MS (24). As in AQP4 ab–positive NMOSD, an algorithmic approach combining several criteria assessable on conventional brain MRIs (lesion adjacent to the body of a lateral ventricle and inferior temporal lobe lesion, U fiber lesion and Dawson's fingers) was able to discriminate between RRMS and MOGAD with good sensitivity and specificity but failed to distinguish MOGAD from AQP4 ab–positive NMOSD (286). These findings were replicated in Korean and Chinese populations in whom a distinction of MS from AQP4 ab–positive NMOSD and MOGAD was achievable with good sensitivity and specificity (287, 288); data on a distinction between MOGAD and AQP4 ab–positive NMOSD were not provided. Using principal component analysis on conventional brain images, another study was also not successful in accurately discriminating MOGAD from AQP ab–positive NMOSD (289).

In contrast, orbital MRI seems to exhibit distinctive features. A combined brain and optic nerve MRI study from Australia in 11 AQP4 ab–positive, 19 MOGAD, and 13 MS patients with a first ON in the investigated eye found more frequent optic nerve swelling in MOGAD and more frequent bilateral optic tract and chiasmal involvement in AQP4 ab–positive NMOSD (205). A predominant affection of the anterior structures of the optic nerve and bilateral involvement were also reported in MOGAD patients from the United States (281).

Retinal OCT findings in MOGAD have been inconclusive. Although some studies suggest that ON in MOGAD causes less severe retinal damage in comparison to AQP4 ab–positive NMOSD (206, 290, 291), others have found comparable thinning of the RNFL and the GCIPL in MOGAD and AQP4 ab–positive NMOSD, probably resulting from the higher ON attack rate in MOGAD (292) (Figure 5). These studies are consistent in suggesting that a single ON episode in MOGAD probably is more benign regarding its effect on the retina than a single ON attack in AQP4 ab–positive NMOSD. This is in line with several other studies reporting a generally favorable outcome from ON in MOGAD; however, exceptions to this rule with poor outcome have also been published (293–296). Interestingly, MOGAD patients seem to have better visual outcomes after ON than AQP4 ab–positive NMOSD despite similar severity of macular GCIPL thinning (297). The issue of subclinical retinal involvement in MOGAD in the absence of ON has not been well-explored. One cross-sectional study found pRNFL thinning in MOGAD NON eyes and an MME prevalence of 26% (298), and one longitudinal study with 38 eyes (18 without ON history, 20 with ON) from 24 MOGAD patients detected a higher rate of annual RNFL thinning than in healthy subjects (299). However, this was not accompanied by progressive GCIPL thinning and the reduction of RNFL over time was driven by a subgroup of patients with thicker RNFL at baseline so the question as to whether progressive retinal thinning occurs in MOGAD requires further investigation.

## Future Directions

Although previous research in advanced neuroimaging led to a tremendous amount of new methods, parameters, and insights into MS and NMOSD diagnostic approaches and pathophysiological processes, further efforts are highly required to make these advances applicable to the clinical setting. Main short- to mid-term aims are (1) standardization of MRI and OCT parameters related to image acquisition and post-processing, (2) transfer and integration of non-conventional techniques into clinically usable procedures, and (3) validation by comparing these readily accessible techniques with current standards within the framework of large patient cohort studies and real-world research. The ultimate goal is to provide the most accurate and most cost- and time-effective markers for clinical diagnostics, therapeutic monitoring, and prognostic forecasting in individual MS and NMOSD patients.

Among the multitude of potential candidates, a selection of promising markers to be introduced into the clinical setting in the near future are the central vein sign at 3 Tesla MRI that has proven to substantially increase specificity of current McDonald 2017 diagnostic criteria in the detection of MS (58), global cerebral and specific regional cortical and deep-gray matter atrophy for monitoring and predicting disease progression and cognitive dysfunction in MS (73), OCT retinal ganglion cell layer thickness as a prognostic marker for future disease activity in patients with clinically isolated syndrome (168), and spinal cord atrophy markers for diagnostic discrimination between AQP4 ab-positive NMOSD and MOGAD and to monitor disease activity in these entities (239). However, strong efforts in terms of observational studies and testing of these markers in clinical trials are necessary to foster their establishment in clinical research and routine.

Because availability of ultra-high field (7 T) scanners has gradually increased during recent years, a noticeable shift of neuroimaging research to higher field strengths will take place in the future. By use of its higher spatial resolution and benefits to imaging contrasts inherent to higher field strengths, 7 T MRI may be used to advance quantitative neuroimaging that may have reached its technical limits at 3 T (131, 300). Moreover, readily accessible 7 T MRI markers, i.e., central vein sign, lesional hypointense rim structures, and gray matter lesion detection might aid to establish accurate diagnoses of MS, especially in patients with conflicting neuroinflammatory disease presentation, when introduced into clinical work-up (52). However, thorough research efforts are necessary to prove potential benefits of ultra-high field MRI compared to conventional MRI in the clinical setting.

Another steadily expanding field of research in MS and NMOSD that will be of interest in the long-term future of neuroimaging, is the emerging application of MRI functional and structural connectome analyses. These techniques provide novel measures by assessing the integrity and functionality of the entire CNS system rather than evaluating separate regional or qualitative alterations in isolation (301). Pathological changes in the functional network integrity in terms of network disruption or even “network collapse” show close correlations to higher order dysfunctions, i.e., predominantly cognitive deficits, in patients with MS (302). Analogously, ON status of CIS and NMOSD patients is associated with decreased connectivity in visual network density as revealed by recent application of graph theory–based tools to analyze functional imaging data (303). These findings are complemented by similar evidence in structural connectome disruption with associations to disease burden in CIS and MS (304). In addition, recent graph theory–based investigations that showed associations between decreased nucleus accumbens and caudate nucleus volumes with higher combined attack type count and longer disease duration in NMOSD lend support to the notion that multimodal network analyses including OCT and MRI parameters may help to identify subsets of promising useful imaging markers (305). However, because validity and potential clinical usefulness of these methods are still unclear, future studies will be undertaken to assess the true capacity of modern neuroimaging connectomics and graph theory–based methods to explain pathological mechanisms and to aid in monitoring and predicting specific disease activity in MS and NMOSD patients.

## Conclusions

Imaging research in autoimmune inflammatory CNS disease has made impressive progress over the past 20 years. Yet, although we are able to deploy structural and functional imaging techniques even in patients at almost subcellular resolution that have significantly contributed to our understanding of mechanisms of tissue damage in these conditions, most of these technologies still await a validated implementation in clinical practice. This, however, is an indispensable prerequisite to make use of these advances to inform treatment decisions and monitor disease activity in individual patients. Because this has remained an unmet need from our patients' perspective, this task will hopefully be tackled despite further thrilling developments in the field of neuroimaging in autoimmune neuroinflammation, for example, OCT angiography, improved post-processing and segmentation techniques, and the use of deep learning and artificial intelligence algorithms (264, 306, 307). Similar endeavors are underway in magnetic resonance imaging that will likely revolutionize our approaches to visualize the brain (128).

## Author Contributions

FP designed the concept of the manuscript, drafted and wrote the work, and critically revised the manuscript. JK assembled, prepared and interpreted imaging data and critically revised, and added to the manuscript regarding important intellectual content.

## Conflict of Interest

The authors declare that the research was conducted in the absence of any commercial or financial relationships that could be construed as a potential conflict of interest. JK received congress registration fees from Biogen, speaker honoraria from Sanofi Genzyme and Bayer Schering, and research support from Krankheitsbezogenes Kompetenznetz, Multiple Sklerose (KKNMS), not related to this work. JK is participant in the BIH-Charité Junior Clinician Scientist Program funded by the Charité-Universitätsmedizin Berlin and Berlin Institute of Health.
